# Learning distributed representations of RNA and protein sequences and its application for predicting lncRNA-protein interactions

**DOI:** 10.1016/j.csbj.2019.11.004

**Published:** 2019-11-30

**Authors:** Hai-Cheng Yi, Zhu-Hong You, Li Cheng, Xi Zhou, Tong-Hai Jiang, Xiao Li, Yan-Bin Wang

**Affiliations:** a The Xinjiang Technical Institute of Physics and Chemistry, Chinese Academy of Sciences, Urumqi 830011, China; b University of Chinese Academy of Sciences, Beijing 100049, China

**Keywords:** Distribution representation, Natural language processing, Word2vec, RNA-protein interaction

## Abstract

The long noncoding RNAs (lncRNAs) are ubiquitous in organisms and play crucial role in a variety of biological processes and complex diseases. Emerging evidences suggest that lncRNAs interact with corresponding proteins to perform their regulatory functions. Therefore, identifying interacting lncRNA-protein pairs is the first step in understanding the function and mechanism of lncRNA. Since it is time-consuming and expensive to determine lncRNA-protein interactions by high-throughput experiments, more robust and accurate computational methods need to be developed. In this study, we developed a new sequence distributed representation learning based method for potential lncRNA-Protein Interactions Prediction, named LPI-Pred, which is inspired by the similarity between natural language and biological sequences. More specifically, lncRNA and protein sequences were divided into *k*-mer segmentation, which can be regard as “word” in natural language processing. Then, we trained out the RNA2vec and Pro2vec model using word2vec and *human* genome-wide lncRNA and protein sequences to mine distribution representation of RNA and protein. Then, the dimension of complex features is reduced by using feature selection based on Gini information impurity measure. Finally, these discriminative features are used to train a Random Forest classifier to predict lncRNA-protein interactions. Five-fold cross-validation was adopted to evaluate the performance of LPI-Pred on three benchmark datasets, including RPI369, RPI488 and RPI2241. The results demonstrate that LPI-Pred can be a useful tool to provide reliable guidance for biological research.

## Graphical abstract

**Figure:**
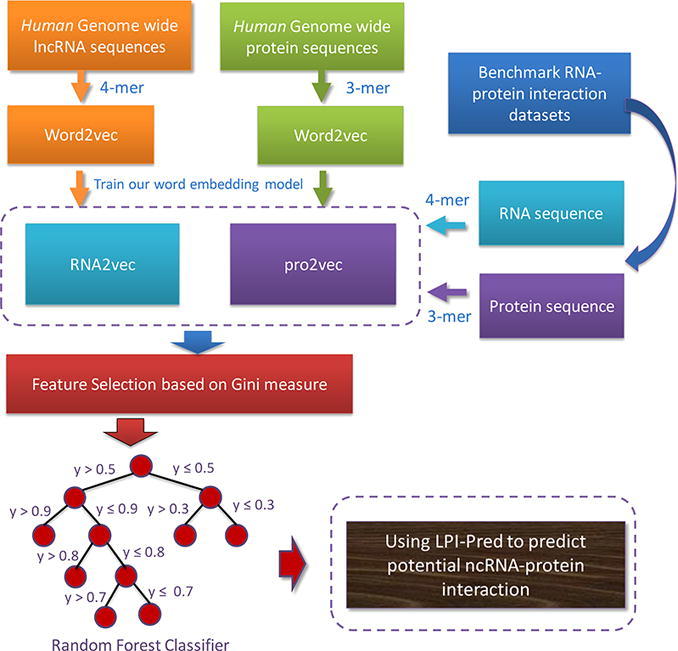

## 1 Introduction

The emerging recognition of RNA is that any transcripts, regardless of protein coding potential, can have intrinsic functions [1]. One kind of this transcripts that are no less than 200 nucleotides, known as long non-coding RNA (lncRNA). Existing studies demonstrate that only less than 2% of the human genome can be translated into proteins, whereas more than 80% of it has biochemical functions [2], [3]. Furthermore, more than 70% of ncRNA are long ncRNA [4], which means there is massive of precious information lncRNAs contained awaiting our effective mining. The lncRNA often act through functions by binding to partner proteins, and play critical roles in gene regulation, splicing, translation, chromatin modification and poly-adenylation [5], [6], [7], [8]. Moreover, emerging evidences have revealed that various complex diseases have strong correlation with lncRNAs, such as Alzheimer [9], lung cancer [10] and cardiovascular diseases [11]. Therefore, the basis for understanding the functions of lncRNA is to identify lncRNA-protein interactions. It’s inefficient to examine a large number of under-researched lncRNAs and proteins though wet experiments.

Due to the time-consuming and laborious of high throughput experiments, such as CLIP-seq, RIP-seq and fRIP-seq [12], several computational lncRNA-protein interaction prediction methods have been put forward in recent years, which can be used as guide tools for biological experiments. These methods can be divided into two categories. The first kind of methods mainly based on sequence information, structural information, evolutionary knowledge or physicochemical properties to exploit discriminative features of lncRNA and protein. For instance, Muppirala et al. proposed RPISeq, which adopted k-mer composition to encode RNA and protein sequences and trained support vector machine (SVM) and Random Forest (RF) model to identify interactions [13]. Suresh et al. used sequence information and structure information to build a SVM predictor to predict novel protein-RNA interactions, named PRI-Pred [14]. Bellucci et al. developed catPAPID by using the physicochemical properties of nucleotide and polypeptide, include secondary structure, Van der Waals propensities and hydrogen bonding, to evaluate the interaction propensities, and they further applied this model to predicted protein interactions in the Xist network [15], [16]. Lu et al. scored RNA-protein pair by using matrix multiplication and Fisher’s linear discriminant. More recently, Yi et al. presented a deep learning framework RPI-SAN, using stacked autoencoder to extract high-level hidden feature from sequence, then they trained RF classifier and ensemble strategy to robustly and accurately predict ncRNA-protein interactions [17]. These methods suggested that the sequence carried enough information for prediction tasks.

There is another category of methods in this domain, which considered the known interactions between lncRNA and protein. Yun et al. considered the relatedness of heterogeneous objects path-constrained, introduced a method using HeteSim measure to compute the relatedness score, called PLPIHS [18]. Zhang et al. using graph regularized nonnegative matrix factorization to discover unknown interacted pairs based on the hypothesis that similar lncRNAs (proteins) have similar corresponding proteins (lncRNAs) [19]. Shen et al. proposed LPI-KTASLP to identify lncRNA-protein interactions with kernel target alignment and semi-supervised link prediction model using multivariate information [20]. Zhang et al. combined multiple sequence-based features and lncRNA-lncRNA similarities and protein-protein similarities, which is calculated by using RNA sequences and protein sequences and known lncRNA-protein interactions [21]. But these kind methods have limitations when predicting new samples, especially those never appeared in the similarity matrices.

This paper aims to develop a new sequence distributed representation learning based method for novel **l**ncRNA-Protein **I**nteractions **Pred**iction, named **LPI-Pred**, which is inspired by the similarity between biological sequences and natural languages [22]. More specifically, lncRNA and protein sequences were divided into *k*-mer segmentation, which can be regard as “word” in natural language processing. Furthermore, we trained the RNA2vec and Pro2vec model using skip-gram word embedding model and *Human* genome-wide lncRNA and protein sequences for lncRNA and protein, respectively. The aforementioned train sequences data are provided by the GENCODE project (release v29) [23]. And then, we measured the importance of features via Gini information impurity, and select top-50 feature as final discriminative features. Finally, these features are used to train RF predictor. We evaluated our model on three benchmark datasets under five-fold cross-validation, including RNA-protein interaction datasets, RPI369 and RPI1807, and lncRNA-protein interaction dataset, RPI488, using six widely used evaluation indicators in machine learning field. And we compared our model with other state-of-the-art models such as RPISeq [13], lncPro [24], and RPI-SAN [17]. The rigorous experimental results prove the validity and reliability of our method.

## 2 Materials and methodology

### 2.1 Datasets exploration

In practice, three benchmark datasets, including RPI369 [13], RPI1807 [14] and RPI488 [25] were selected to execute our evaluation. The first two are RNA-protein interactions datasets, while the third is lncRNA-protein interactions dataset. The RPI369 dataset is a non-redundant data set, which is generated from RPIDB [26], and only have non-ribosomal complexes (e.g., mRNA, miRNA, tRNA). The dataset RPI369 contains 332 RNA sequences, 338 protein sequences and 369 positive interaction pairs. In the same work, the authors also constructed another dataset RPI2241, which is larger than RPI369 but is strongly biased to ribosomal RNA-protein interactions. That's why we're not inclined to adopt it. The RPI1807 also is a non-redundant data set of RNA-protein interactions complexes, generated by parsing the RPIDB and Nucleic Acid Database (NDB) [24]. There are 1078 RNA sequences and 1807 protein sequences in RPI1807, consisting 1807 pairs positive samples and 1436 pairs negative samples. The RPI488 is a lncRNA-protein interactions dataset, contains 245 negative lncRNA-protein pairs, 243 interacted lncRNA-protein pairs. The number of lncRNA and protein in this dataset are 25 and 247, respectively. The details of these three benchmark datasets are listed in Table 1 as below:

**Table 1:** The details of two RNA-protein interactions datasets RPI369 and RPI1807 and lncRNA-protein interactions dataset RPI488.

Datasets	# of RNAs	# of proteins	Positive samples	Negative samples	References
RPI369	332	338	369	369	[13]
RPI1807	1078	1807	1807	1807	[14]
RPI488	25	247	243	245	[25]

### 2.2 *k*-mer segmentation

**Fig. 1:**
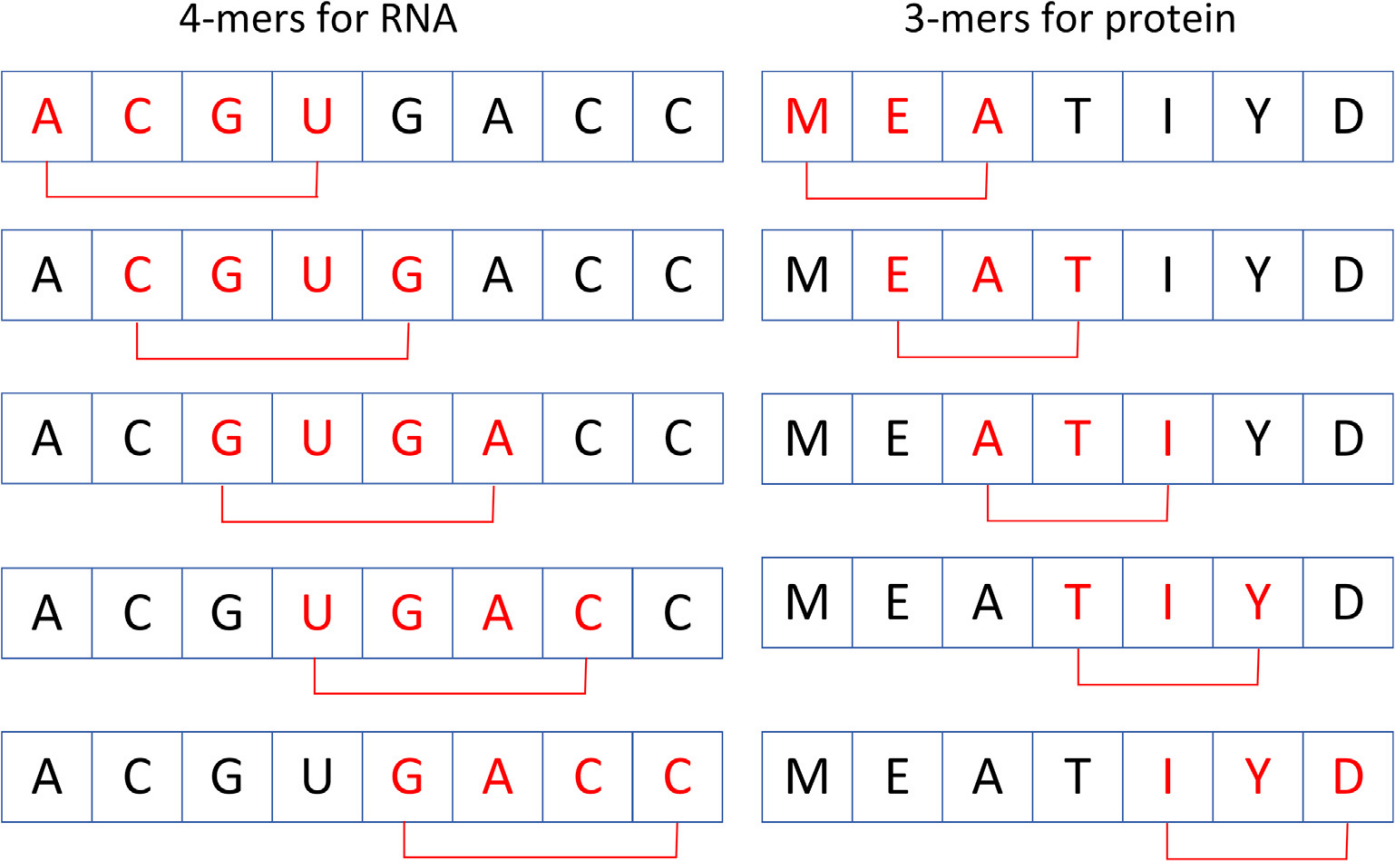
Procedure of splitting RNA nucleotides and protein amino acids sequences into smaller *k*-mers.

In this section, we will introduce the feature representation scheme used in this study, which is aims to fully exploit the hidden high-level feature from the sequence information. For a given lncRNA or protein sequence, *k*-mer composition is used to spilt them into subsequences, which can be considered as “word” in the fellow step. Scan a sequence from beginning to the end, one nucleic acid once time. For a given sequence of length *L*, we will obtain L-k+1 *k*-mers, and the count of possible *k*-mer are $4^{k}$ for RNA (A, C, G, U) and $20^{k}$ for protein (Ala, Gly, Val, Ile, Leu, Phe, Pro, Tyr, Met, Thr, Ser, His, Asn, Gln, Tpr, Arg, Lys, Asp, Glu, Cys), different from common usage, we do not use the 7-letter reduce alphabet, which reduced 20 amino acids into 7 groups based on their similarity of dipole moments and side chain volume. We set the *k* to 4 for lncRNA and set *k* to 3 for protein, which are two commonly accepted empirical parameters [13], [17], [25], [27]. The process of splitting nucleic acids sequence and amino acids sequences into *k*-mers shown in Fig. 1.

### 2.3 Distribution representation of lncRNA and protein sequences

**Fig. 2:**
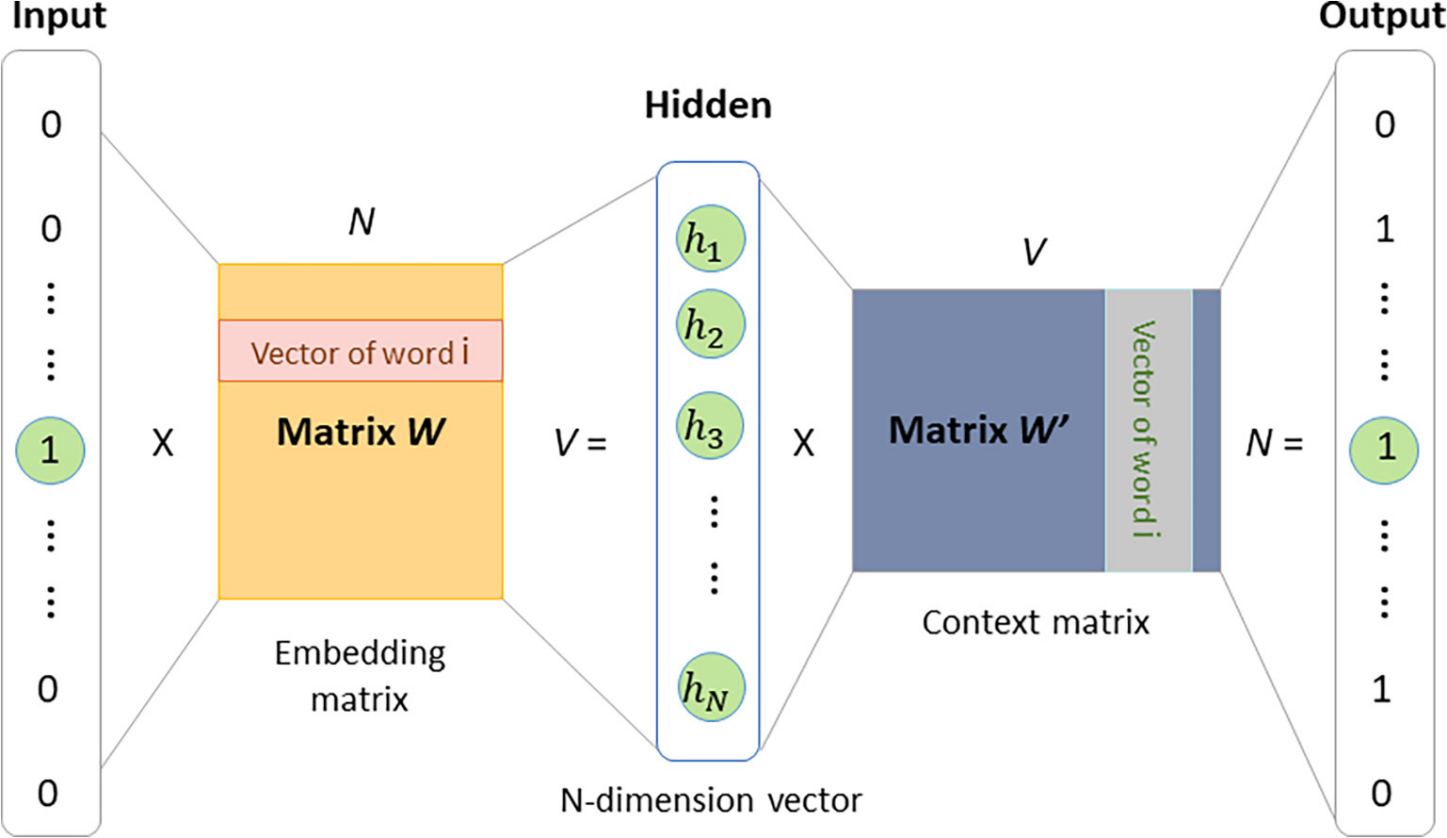
The skip-gram word embedding model. Lnc2vec and pro2vec model were trained by using this model and genome-wide human lncRNA and protein sequences. Skip-gram is trained by predicting words surrounding the central word, after training, the weights matrix *W* of the hidden layer is obtained, that is word vectors.

And then, we using the genome-wide human lncRNA and protein sequences to train a word embedding model, named RNA2vec and pro2vec, respectively. The training data provided by the GENCODE project and their goal of this project is to identify and classify all gene features in the human and mouse genomes with high accuracy based on biological evidence, and to release these annotations [23], [28]. We use the skip-gram [29], [30] word representation model to learn distribution representation of RNA and protein sequences. In nature, the model is a neural network with projection layer for learning word representation. The structure of skip-gram is shown in Fig. 2 below.

For a given sequence ($w_{1}$, $w_{2}$, …, $w_{l-k+1}$), the goal of training model is to maximize the mean log probability:

(1) $$\max\frac{1}{N}\sum_{n=1}^{N}\sum_{-c\leq m\leq c,m\neq 0}logP\left(w_{n+m}|w_{n}\right)$$

*c* stands for the distance to the central word; the log probability distribution can be defined as follow:

(2) $$\log P\left(w_{o}|w_{i}\right)=log\frac{e^{{v\text{'}}_{w_{o}}^{T}v_{w_{i}}}}{\sum_{w=1}^{W}e^{{v\text{'}}_{w}^{T}v_{w_{i}}}}$$

where the $v_{w}$ and ${v\text{'}}_{w}$ are the input and output vector of word w, respectively. *W* is the size of training lncRNA or protein training lexicon.

**Fig. 3:**
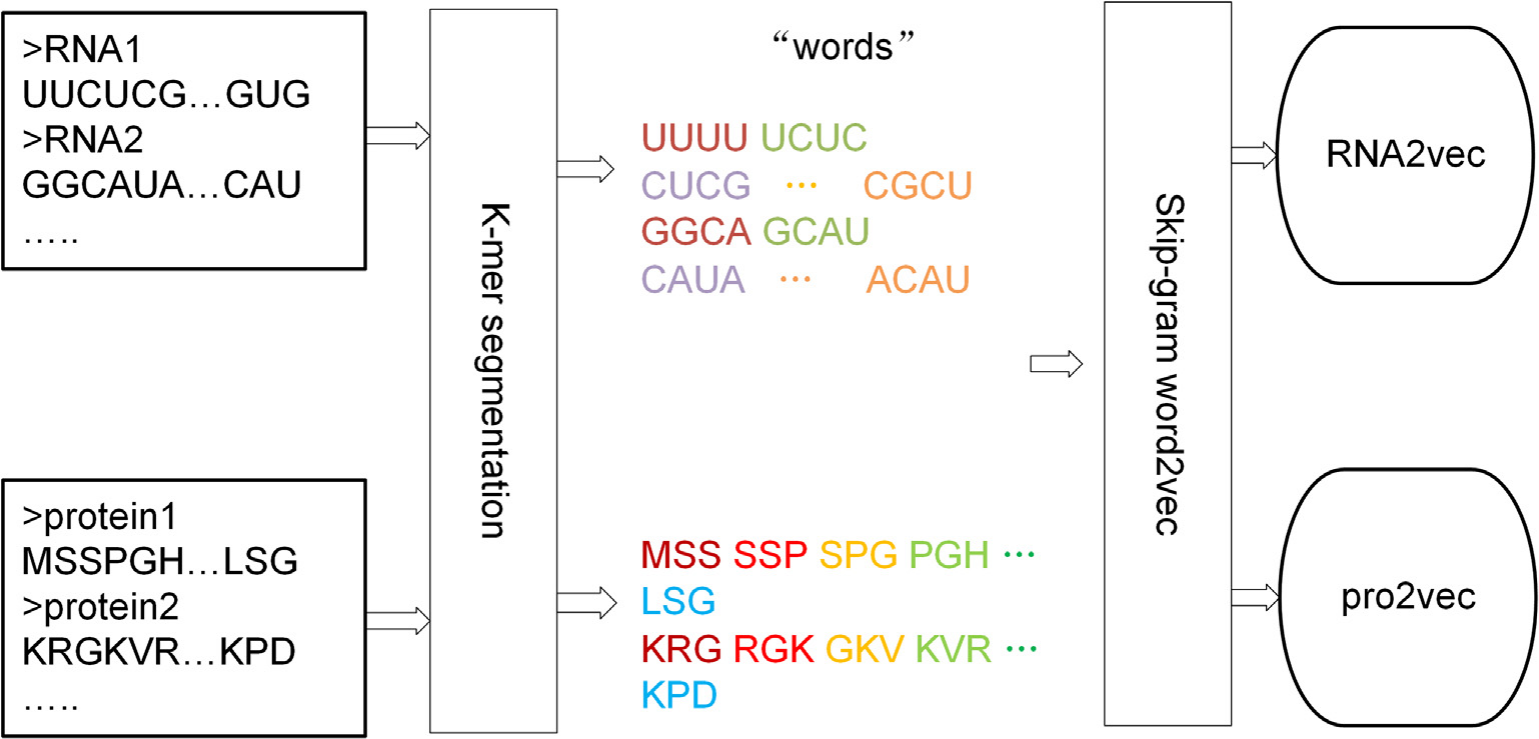
**.** The procedure for training RNA2vec and pro2vec. The corpus of RNA and protein sequences obtained from GENCODE project. And the model implemented by word2vec.

In natural language processing, the word embedding model has achieved great success [31], [32], it has also made progress in computational biology [33], [34], [35]. In this work, we regard each k-mer as a word and a sequence as a sentence, and then learning the distribution representation by using skip-gram word2vec model. The procedure for training RNA2vec and pro2vec is shown as Fig. 3.

The parameters of the model are *min_count* = 1, *size* = 300, *window* = 5, *iter* = 10, *batch_words* = 100. Where the *size* represents the dimensions of output word vector, and *window* stands for maximum distance between the current and predicted word within a sentence, *iter* is the count of iterations (epochs) over the corpus, *batch_words* is the target size (in words) for batches of examples passed to worker threads. When the *min_count* (means minimum word frequency) is set too high, the model only counts high-frequency words, which is not conducive to learning discriminative word vectors from sequence representation. Other parameters are default. Inspired by the additivity of word embedding [30], we represented a given sequence by summing all its *k*-mer word embeddings. Here, we obtained the word embedding feature as base feature. The procedure for training RNA2vec and pro2vec is shown as Fig. 3.

### 2.4 Gini information impurity-based feature selection

A data set often has hundreds of previous features. How to choose the features that have the greatest impact on the results, so as to reduce the number of features when building the model. There are many such methods, for instance, principal component analysis, Lasso [36], [37], mRMR [38] and so on. However, here we are going to introduce the use of Random Forest to feature screening based on Gini information impurity.

Assuming that there are *m* features $f_{1}$, $f_{2}$, $f_{\cdots }$, $f_{m}$, we can calculate the Variable Importance Measures (*VIM*) by the Gini index ${\mathrm{VIM}}_{i}^{\left(Gini\right)}$ for each feature $f_{i}$, that is, the average change of node splitting impurity in all RF decision trees by $f_{i}$ feature. The Gini index (*GI*) can be defined as:

(3) $${\mathrm{GI}}_{i}=\sum_{k=1}^{\left|K\right|}\sum_{\underset{\text{'}}{k}\neq k}p_{\mathrm{ik}}p_{ik^{\text{'}}}=1-\sum_{k=1}^{\left|K\right|}p_{\mathrm{ik}}^{2}$$

where the *K* means there are *k* categories, and $p_{\mathrm{ik}}$ indicates the proportion of categories *k* in $i_{\mathrm{th}}$ node. The *VIM* of feature $f_{i}$ in $j_{\mathrm{th}}$ node can be computed from the variation of *GI* before and after branching of $j_{th}$ node:

(4) $${\mathrm{VIM}}_{\mathrm{ij}}^{\left(Gini\right)}={\mathrm{GI}}_{i}-{\mathrm{GI}}_{r}-{\mathrm{GI}}_{l}$$

Among them, ${\mathrm{GI}}_{r}$ and ${\mathrm{GI}}_{l}$ respectively represent the *GI* of the right and left nodes after branching. Suppose there are *N* decision trees, so:

(5) $${\mathrm{VIM}}_{i}^{\left(Gini\right)}=\sum_{n=1}^{N}{\mathrm{VIM}}_{\mathrm{ij}}^{\left(Gini\right)}$$

Finally, all the obtained importance scores can be normalized by:

(6) $${\mathrm{VIM}}_{i}=\frac{{\mathrm{VIM}}_{i}}{\sum_{j=1}^{c}{\mathrm{VIM}}_{j}}$$

Here, we selected the most important top-50 features as final feature.

### 2.5 Training an LPI-Pred model

**Fig. 4:**
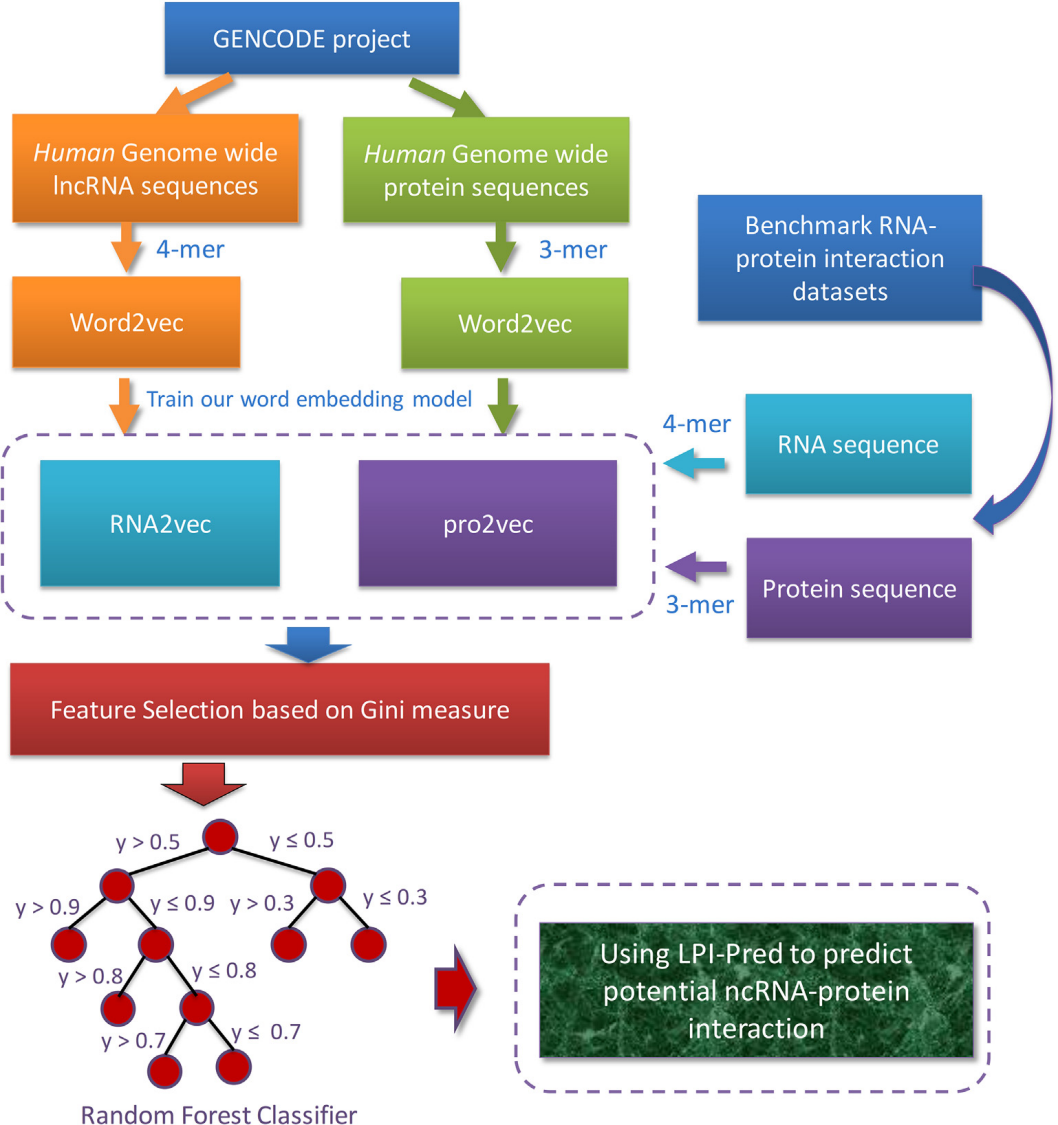
The workflow of LPI-Pred. The word embedding model RNA2vec and pro2vec are trained to obtain the sequence information of RNA and protein, and these features after feature selection are used to train Random Forest predictor.

The selected top-50 feature would be used to train an LPI-Pred model for predicting potential lncRNA-protein interactions on test data set. In summary, the procedure for training an LPI-Pred is shown in Fig. 4:

• Using *human* genome-wide lncRNA and protein sequences as corpus, segment them into *k*-mers as the words.
• Using word2vec model to train out RNA2vec and pro2vec for lncRNA and protein sequence distribution representation.
• Obtaining the word embedding of the protein and ncRNA sequences in the benchmark RNA-protein interaction datasets.
• Select top-50 features based on feature importance to train Random Forest predictor.

### 2.6 Performance evaluation metrics

In this study, we proposed a novel lncRNA-protein interactions prediction model LPI-Pred, based on sequence distributed representation learning and Gini information impurity measure. The common metrics and five-fold cross-validation are used to evaluate the performance of LPI-Pred. Divided all data into five equal sub-set. For each training, one-fold set data is taken as test data, the rest four-fold are taken as training data. Take the mean performance metrics of five training as final performance. There is no overlap between train data and test data, and this is unbiased comparison. The metrics used in performance evaluation including accuracy (Acc), Sensitivity (Sens), Specificity (Spec), Precision (Pre) and Matthews Correlation Coefficient (MCC). Certainly, and the area under the curve (AUC) of the Receiver Operating Characteristic (ROC) curve are also adopted to evaluate the performance. These metrics can be defined as:

(7) $$Acc=\frac{\mathrm{TN}+TP}{\mathrm{TN}+TP+FN+FP}$$

(8) $$Sensitivity=\frac{\mathrm{TP}}{\mathrm{TP}+FN}$$

(9) $$Specificity=\frac{\mathrm{TN}}{\mathrm{TN}+FP}$$

(10) $$Precision=\frac{\mathrm{TP}}{\mathrm{TP}+FP}$$

(11) $$MCC=\frac{\mathrm{TP}\times TN-FP\times FN}{\sqrt{\left(TP+FP)(TP+FN)(TN+FP)(TN+FN\right)}}$$

where *TN, TP* indicates the correctly predicted negative samples and positive samples number, *FN*, *FP* represents the false wrongly predicted negative and positive samples number.

## 3 Results and discussion

In this study, we proposed a novel lncRNA-protein interactions prediction model LPI-Pred, based on sequence distributed representation learning and Gini information impurity measure. In this section, we designed the following experiments to verify the performance of the model. First, we compared the effects of different sequence coding schemes on lncRNA-protein interaction dataset, and the effect of feature selection. Second, we did a performance comparison with different individual predictors. And then, we verify LPI-Pred’s ability to predict lncRNA-protein interactions and compared with other state-of-the-art methods. Final, we apply our model to lncRNA-protein interactions network construction.

### 3.1 Comparison between different sequences encoding strategies

We applied a new RNA and protein sequences encoding method in this work, using skip-gram distribution representation model. In order to verify the effectiveness of this sequence numerical coding scheme, we first compare it with the widely used *k*-mer frequency on three benchmark datasets. The comparison results are shown in Table 2.

**Table 2:** Comparing the five-fold cross-validation performance of *k*-mer and word embedding with and without feature selection on three gold standard datasets.

Datasets	feature	Acc (%)	Sens (%)	Spec (%)	Pre (%)	MCC (%)
RPI369	*k*-mer	68.71	67.29	70.30	69.88	37.74
	embedding without feature selection	71.97	70.27	**73.76**	**73.19**	44.24
	embedding with feature selection	**73.06**	**75.32**	71.14	72.64	**46.67**
RPI488	*k*-mer	89.29	**83.17**	95.17	94.33	79.09
	embedding without feature selection	87.64	83.17	91.93	90.82	75.52
	embedding with feature selection	**89.92**	82.75	**96.72**	**96.32**	**80.59**
RPI1807	*k*-mer	96.88	**98.44**	94.96	96.04	93.72
	embedding without feature selection	96.73	97.90	95.28	96.28	93.37
	embedding with feature selection	**97.10**	97.89	**96.14**	**96.91**	**94.13**

The boldface indicates this measure performance is the best among the compared sequence feature encoding.

In all three gold standard datasets, the selected word embedding feature, obtained though RNA2vec and pro2vec model, have improved performance compared to *k*-mer method. This can prove that distribution representation word vector is effectiveness for biological sequences encoding, for RNA and protein. It can achieve and even exceed the performance of *k*-mer, which is very widely used in biological sequence representation. The comparison between LPI-Pred (using RNA2vec and pro2vec with feature selection) and LPI-Pred without feature selection demonstrate the necessity of feature selection.

### 3.2 Comparison with individual predictors

To verify the effect of RF classifier separately, we compared RF and other machine learning modals including SVM (with RBF kernel), Logistic Regression (LR), under same set of features and the same experimental conditions. These models were trained with default parameters. The results are shown in Table 3:

**Table 3:** Comparing the five-fold cross-validation performance of LPI-Pred and other machine learning classifiers on three gold standard datasets.

Datasets	Methods	Acc (%)	Sens (%)	Spec (%)	Pre (%)	MCC (%)
RPI369	SVM	65.17	66.20	64.34	65.48	30.61
	LR	58.37	44.06	**73.12**	62.51	18.05
	LPI-Pred	**73.06**	**75.32**	71.14	**72.64**	**46.67**
RPI488	SVM	88.68	81.97	95.17	94.26	77.95
	LR	88.68	81.97	95.17	94.26	77.95
	LPI-Pred	**89.92**	**82.75**	**96.72**	**96.32**	**80.59**
RPI1807	SVM	92.35	94.11	90.17	92.29	84.52
	LR	87.26	90.17	83.56	87.39	74.17
	LPI-Pred	**97.10**	**97.89**	**96.14**	**96.91**	**94.13**

The boldface indicates this measure performance is the best among the compared methods for individual dataset.

Several Random Forest-based methods have achieved remarkable performance on many issues in the field of computational biology. We trained LPI-Pred based on random forest classifier. As shown in the comparison results in the above table, LPI-Pred outperformed all other classifiers using same feature set and under same experimental conditions.

### 3.3 Evaluation of LPI-Pred's capability to predict lncRNA-protein interactions

Furthermore, we compared our model with other state-of-the-art methods including RPISeq [13], lncPro [24], and RPI-SAN [17] to evaluate the predictive ability to lncRNA-protein interactions of LPI-Pred. The RPISeq and lncPro use only sequence information, which is similar to LPI-Pred. More recently, the RPI-SAN use deep learning model, based on sequence information and evolutionary information to predict novel ncRNA-protein interactions. We follow same performance evaluation measurements. The comparison details are shown as below Table 4.

**Table 4:** Comparing five-fold cross-validation performance of LPI-Pred and other state-of-the-art methods on three gold standard datasets.

Datasets	Methods	Acc (%)	Sens (%)	Spec (%)	Pre (%)	MCC (%)	AUC
RPI369	RPISeq	70.4	70.5	70.2	70.7	40.9	0.767
	lncPro	70.4	70.8	69.6	71.3	40.9	0.740
	LPI-Pred	**73.06**	**75.32**	**71.14**	**72.64**	**46.67**	**0.802**
RPI1807	RPISeq	**97.3**	96.8	98.4	96.0	**94.6**	0.996
	lncPro	96.9	96.5	98.1	95.5	93.8	0.994
	RPI-SAN	96.1	93.6	**99.9**	91.4	92.4	**0.999**
	LPI-Pred	97.10	**97.89**	96.14	**96.91**	94.13	0.994
RPI488	RPISeq	88.0	92.6	82.2	93.2	76.2	0.903
	lncPro	87.0	90.0	82.7	91.0	74.0	0.901
	RPI-SAN	89.7	**94.3**	83.7	95.2	79.3	**0.920**
	LPI-Pred	**89.92**	82.75	**96.72**	**96.32**	**80.59**	0.911

The boldface indicates this measure performance is the best among the compared methods for individual dataset.

On dataset RPI369, LPI-Pred performs better than RPISeq and lncPro on all measurements, with accuracy of 73.06%, sensitivity of 75.32%, specificity of 71.14%, precision of 72.64%, MCC of 46.67% and AUC of 0.802. For dataset RPI1807, LPI-Pred is not best on all 6 indicators, but it still has an accuracy of up to 97.1%, and perform better on sensitivity and precision. Essentially, the RPI488 is the full lncRNA-protein interactions dataset. As the results shown, the accuracy, sensitivity, specificity, precision, MCC and AUC of LPI-Pred are 89.92%, 82.75%, 96.72%, 96.32%, 80.59% and 0.911. It has the best performance on accuracy, specificity, precision and MCC compared with all existing methods. Overall, the evaluation between LPI-Pred and other methods on three benchmark datasets can prove the high robustness and accuracy of LPI-Pred. It suggests that the word embedding can provide hidden high-level feature of sequence and the feature selection can further enhance the expressiveness of features and reduce the complexity of model training.

## 4 Conclusion

The lncRNA-protein interactions play numerous roles in life activities, cellular function and disease. The first step in studying its function and mechanism is to identify interacting lncRNA-protein pairs. In this study, we present a novel lncRNA-protein interaction prediction model named LPI-Pred. First, we trained distribution representation model, RNA2vec and pro2vec, by using skip-gram word embedding model and *human* genome-wide lncRNA and protein sequences. Then, we convert the lncRNA and protein sequence into word vector using the model trained above. The Gini impurity-based feature selection is used to obtain discriminative features. Then we training LPI-Pred to predict lncRNA-protein interactions. We compared the performance of different feature representations and predictors, and we also compared LPI-pred with other state-of-the-art methods. The rigorous evaluation experimental results show the effectiveness and robustness of our model.

Inspired by the similarity between biological sequences and natural language sentences, we divided sequence into *k*-mers, which can be considered as “words” in biological language. The experimental proved this feature extraction scheme works well. However, rethinking of the procedure of RNA2vec and pro2vec, we recognize that *k*-mer may not be the best way to sequence word segmentation. More bio-semantic sequence segmentation should be explored in the future.

## Author contributions

H-C. Y and Z-H. Y conceived the algorithm, carried out analyses, prepared the data sets, carried out experiments, and wrote the manuscript; L. C, X. Z, T-H. J and X. L designed, performed and analyzed experiments and wrote the manuscript; All authors read and approved the final manuscript.

## Declaration of Competing Interest

The authors declare that they have no known competing financial interests or personal relationships that could have appeared to influence the work reported in this paper.

## References

[1] Han S, Du W, Xu Y, Zhang Y, Li Y, Liang Y, Ma Q, Wang C: LncFinder: an integrated platform for long non-coding RNA identification utilizing sequence intrinsic composition, structural information and physicochemical property. 2018. 10.1093/bib/bby065 PMC6954391 30084867

[2] Djebali S., Davis C.A., Merkel A., Dobin A., Lassmann T., Mortazavi A. (2012). Landscape of transcription in human cells. Nature.

[3] Pennisi E. (2012). ENCODE project writes eulogy for junk DNA. Science.

[4] Yang Q., Zhang S., Liu H., Wu J., Xu E., Peng B. (2014). Oncogenic role of long noncoding RNA AF118081 in anti-benzo[a]pyrene-trans-7,8-dihydrodiol-9,10-epoxide-transformed 16HBE cells. Toxicol Lett.

[5] Tsai M.-C., Manor O., Wan Y., Mosammaparast N., Wang J.K., Lan F. (2010). Long noncoding RNA as modular scaffold of histone modification complexes. Science.

[6] Nie L., Wu H.-J., Hsu J.-M., Chang S.-S., Labaff A.M., Li C.-W. (2012). Long non-coding RNAs: versatile master regulators of gene expression and crucial players in cancer. Am J Transl Res.

[7] Zeng X., Lin W., Guo M., Zou Q. (2017). A comprehensive overview and evaluation of circular RNA detection tools. PLoS Comput Biol.

[8] Wang Kevin C., Chang Howard Y. (2011). Molecular mechanisms of long noncoding RNAs. Mol Cell.

[9] Ng S.-Y., Lin L., Soh B.S., Stanton L.W. (2013). Long noncoding RNAs in development and disease of the central nervous system. Trends Genet.

[10] Shi X., Sun M., Liu H., Yao Y., Kong R., Chen F. (2015). A critical role for the long non-coding RNA GAS5 in proliferation and apoptosis in non-small-cell lung cancer. Mol Carcinog.

[11] Congrains A., Kamide K., Oguro R., Yasuda O., Miyata K., Yamamoto E. (2012). Genetic variants at the 9p21 locus contribute to atherosclerosis through modulation of ANRIL and CDKN2A/B. Atherosclerosis.

[12] Colantoni A., Ferrè F., Helmer-Citterich M. (2015). Revealing protein–lncRNA interaction. Briefings Bioinf.

[13] Muppirala U.K., Honavar V.G., Dobbs D. (2011). Predicting RNA-protein interactions using only sequence information. BMC Bioinf.

[14] Suresh V., Liu L., Adjeroh D., Zhou X. (2015). RPI-Pred: predicting ncRNA-protein interaction using sequence and structural information. Nucleic Acids Res.

[15] Bellucci M., Agostini F., Masin M., Tartaglia G.G. (2011). Predicting protein associations with long noncoding RNAs. Nat Methods.

[16] Agostini F., Cirillo D., Bolognesi B., Tartaglia G.G. (2012). X-inactivation: quantitative predictions of protein interactions in the Xist network. Nucleic Acids Res.

[17] Yi H.-C., You Z.-H., Huang D.-S., Li X., Jiang T.-H., Li L.-P. (2018). A deep learning framework for robust and accurate prediction of ncRNA-protein interactions using evolutionary information. Mol Ther Nucleic Acids.

[18] Xiao Y., Zhang J., Deng L. (2017). Prediction of lncRNA-protein interactions using HeteSim scores based on heterogeneous networks. Sci Rep.

[19] Zhang T., Wang M., Xi J., Li A. (2018). LPGNMF: predicting long non-coding RNA and protein interaction using graph regularized nonnegative matrix factorization. IEEE/ACM Trans Comput Biol Bioinf.

[20] Shen C., Ding Y., Tang J., Jiang L., Guo F. (2019). LPI-KTASLP: Prediction of LncRNA-Protein Interaction by Semi-Supervised Link Learning With Multivariate Information. IEEE Access.

[21] Zhang W, Yue X, Guifeng T, Wu W, Huang F, Zhang X: SFPEL-LPI: sequence-based feature projection ensemble learning for predicting LncRNA-protein interactions, 14; 2018. 10.1371/journal.pcbi.1006616 PMC6331124 30533006

[22] Wang Y., You Z.-H., Yang S., Li X., Jiang T.-H., Zhou X. (2019). A high efficient biological language model for predicting protein-protein interactions. Cells.

[23] Frankish A., Bignell A., Berry A., Yates A., Parker A., Schmitt B.M. (2018). GENCODE reference annotation for the human and mouse genomes. Nucleic Acids Res.

[24] Lu Q., Ren S., Lu M., Zhang Y., Zhu D., Zhang X. (2013). Computational prediction of associations between long non-coding RNAs and proteins. BMC Genomics.

[25] Pan X., Fan Y.X., Yan J., Shen H.B. (2016). IPMiner: hidden ncRNA-protein interaction sequential pattern mining with stacked autoencoder for accurate computational prediction. BMC Genomics.

[26] Lewis B.A., Walia R.R., Terribilini M., Ferguson J., Zheng C., Honavar V. (2010). PRIDB: a protein–RNA interface database. Nucleic Acids Res.

[27] Shen J., Zhang J., Luo X., Zhu W., Yu K., Chen K. (2007). Predicting protein–protein interactions based only on sequences information. Proc Natl Acad Sci.

[28] Harrow J., Frankish A., Gonzalez J.M., Tapanari E., Diekhans M., Kokocinski F. (2012). GENCODE: the reference human genome annotation for the ENCODE project. Genome Res.

[29] Le Q, Mikolov T. Distributed representations of sentences and documents. In: International conference on machine learning: 2014. 1188–1196.

[30] Gittens A, Achlioptas D, Mahoney MW: Skip-gram-zipf+ uniform= vector additivity. In: Proceedings of the 55th Annual Meeting of the Association for Computational Linguistics (Volume 1: Long Papers): 2017. 69–76.

[31] Mikolov T., Sutskever I., Chen K., Corrado G.S., Dean J. (2013.). Distributed representations of words and phrases and their compositionality. Adv Neural Inf Process Syst.

[32] Mikolov T, Chen K, Corrado G, Dean J: Efficient estimation of word representations in vector space. arXiv preprint arXiv:13013781 2013.

[33] Asgari E., Mofrad M.R. (2015). Continuous distributed representation of biological sequences for deep proteomics and genomics. PLoS ONE.

[34] Pan X., Shen H.-B. (2018). Learning distributed representations of RNA sequences and its application for predicting RNA-protein binding sites with a convolutional neural network. Neurocomputing.

[35] Wang L., You Z.-H., Chen X., Li Y.-M., Dong Y.-N., Li L.-P. (2019). LMTRDA: Using logistic model tree to predict MiRNA-disease associations by fusing multi-source information of sequences and similarities. PLoS Comput Biol.

[36] Kim S.-J., Koh K., Lustig M., Boyd S., Gorinevsky D. (2007). An interior-point method for large-scale $\ell_1 $-regularized least squares. IEEE J Sel Top Signal Process.

[37] Friedman J., Hastie T., Tibshirani R. (2010). Regularization paths for generalized linear models via coordinate descent. J Stat Softw.

[38] Peng H., Long F., Ding C. (2005). Feature selection based on mutual information: criteria of max-dependency, max-relevance, and min-redundancy. IEEE Trans Pattern Anal Mach Intell.

